# The glycerol-3-phosphate acyltransferase PLAT2 functions in the generation of DHA-rich glycerolipids in *Aurantiochytrium limacinum* F26-b

**DOI:** 10.1371/journal.pone.0211164

**Published:** 2019-01-30

**Authors:** Eri Nutahara, Eriko Abe, Shinya Uno, Yohei Ishibashi, Takashi Watanabe, Masahiro Hayashi, Nozomu Okino, Makoto Ito

**Affiliations:** 1 Department of Bioscience and Biotechnology, Graduate School of Bioresource and Bioenvironmental Sciences, Kyushu University, Moto-oka, Nishi-ku, Fukuoka, Japan; 2 Department of Marine Biology and Environmental Sciences, Faculty of Agriculture, University of Miyazaki, 1–1 Gakuen-Kibanadai-Nishi, Miyazaki, Japan; 3 Innovative Bio-architecture Center, Kyushu University, Moto-oka, Nishi-ku, Fukuoka, Japan; University of Illinois, UNITED STATES

## Abstract

Thraustochytrids possess docosahexaenoic acid (DHA, 22:6n-3) as acyl chain(s) of triacylglycerol (TG) and phosphatidylcholine (PC), some of which contain multiple DHAs. However, little is known about how these DHA-rich glycerolipids are produced in thraustochytrids. In this study, we identified PLAT2 in *Aurantiochytrium limacinum* F26-b as a glycerol-3-phosphate (G3P) acyltransferase (GPAT) by heterologous expression of the gene in budding yeast. Subsequently, we found that GPAT activity was reduced by disruption of the PLAT2 gene in *A*. *limacinum*, resulting in a decrease in DHA-containing lysophosphatidic acid (LPA 22:6). Conversely, overexpression of PLAT2 increased both GPAT activity and LPA 22:6. These results indicate that PLAT2 is a GPAT that transfers DHA to G3P *in vivo* as well as *in vitro*. Overexpression of the PLAT2 gene increased the production of a two DHA-containing diacylglycerol (DG 44:12), followed by an increase in the three DHA-containing TG (TG 66:18), two-DHA-containing TG (TG 60:12), and two DHA-containing PC (PC 44:12). However, overexpression of PLAT2 did not increase DHA-free DG (DG32:0), which was preferentially converted to three 16:0-containing TG (TG 48:0) but not two 16:0-containing PC (PC 32:0). Collectively, we revealed that DHA-rich glycerolipids are produced from a precursor, LPA 22:6, which is generated by incorporating DHA to G3P by PLAT2 in the *A*. *limacinum*.

## Introduction

N-3 polyunsaturated fatty acids (n-3 PUFAs), such as docosahexaenoic acid (DHA, 22:6n-3), decrease the level of blood neutral fat and function in preventing arteriosclerosis and thrombus formation, thereby reducing the risk of cardiovascular diseases [1, 2]. DHA, which is abundant in the brain and retina, is important for the development of the nervous system, the transmission of optical information, and the regeneration of rhodopsin [3, 4]. Recently, DHA was found to alleviate the progression of Alzheimer's disease and suppress neural cell death [5]. In addition, protectin D1 and resolvin D1, metabolites of DHA, have been reported to inhibit the replication of influenza virus [6] and to exhibit anti-inflammatory effects [7]. Based on these health benefits, DHA is used in supplements and medicine, and its demand is rapidly increasing. DHA is industrially produced mainly from fish oils. However, due to the declining fish stocks and yearly fluctuation, an alternative resource is strongly desired for DHA production [8].

Thraustochytrids, eukaryotic single-cell protists belonging to Stramenopiles, live in marine and brackish water environments, especially in the mangrove forest and estuary areas of tropical to subtropical areas [9]. They are heterotrophs that do not perform photosynthesis due to the lack of chloroplasts. Thraustochytrids, like other marine microbes, are considered to be a primary DHA producer in marine environments [10]. Thraustochytrids have suitable properties for industrial production of DHA, i.e., they can be mass cultured using a Jar fermenter without sunlight, and they can accumulate large amounts of DHA in well-developed intracellular lipid droplets (LDs) [11, 12].

Two biosynthesis pathways of DHA have been elucidated in thraustochytrids. *Aurantiochytrium* (formerly *Schizochytrium*) has a polyketide-like synthase pathway in which DHA is directly produced from acetyl-CoA and malonyl-CoA by a multiple enzyme complex, PUFA synthase [13]. On the other hand, *Thraustochytrium aureum* produces DHA not only via PUFA synthase, but also through an elongase/desaturase pathway [14, 15]. Recently, it was reported that *Aurantiochytrium* and related genus have several (but not all) genes involved in elongase/desaturase pathway; however, the biological significance of these genes remains unknown [16, 17]. *De novo* synthesized DHA is incorporated to glycerolipids, such as triacylglycerol (TG) and phosphatidylcholine (PC), as an ester-linked acyl chain(s), and then accumulated in LDs and cell membranes [18–20].

*A*. *limacinum* used in this study possesses DHA-rich TG and PC such as TG 60:12 (22:6/22:6/16:0, two DHAs/molecule), TG 66:18 (22:6/22:6/22:6, three DHAs/molecule), and PC 44:12 (22:6/22:6, two DHAs/molecule) [20]. These DHA-rich glycerolipids are characteristic to thraustochytrids, but not other marine microorganisms belonging to Stramenopiles such as diatoms. The TG content of diatoms, *Thalassiosira pseudonana* and *Phaeodactylum tricornutum*, is about 14–18% of the total dry weight, and TG contains n-3PUFA such as EPA and DHA (mainly EPA) [21, 22]. However, in contrast to *A*. *limacinum*, the content of multiple n-3PUFA-containing TG is very low, and thus the total n-3PUFA level is relatively lower than that of thraustochytrids. The purpose of this study is to clarify how DHA-rich glycerolipids are produced in thraustochytrids.

Recent advances in molecular tools applicable to thraustochytrids have enabled the analysis of lipid metabolism at the molecular level and genetic transformation of thraustochytrids [23–26]. One of the most important issues for obtaining useful transformants by genetic transformation is to identify the limiting factor for the synthesis of DHA-rich glycerolipids.

We report in this paper that DHA-rich glycerolipids are produced from DHA-containing lysophosphatidic acid (LPA 22:6) as a precursor, which is generated by incorporating DHA to glycerol-3-phosphate (G3P) by PhosphoLipid AcylTransferase 2 (PLAT2) in the *A*. *limacinum* F26-b.

This is the first report to describe the molecular mechanism by which DHA-rich glycerolipids are produced in the thraustochytrid, which is a promising industrial as well as model microorganism for the production of DHA.

## Materials and methods

### Materials

Thraustochytrid strain F26-b was isolated from fallen leaves of *Rhizophora mucronata* collected at Ishigaki Is., Okinawa, Japan, and identified as *Aurantiochytrium limacinum* based on 18S rRNA gene analysis and the microscopic morphological features [19]. All cold acyl-CoAs were purchased from Avanti Polar Lipids (Alabaster, AL) and [1-^14^C]palmitoyl-CoA (0.1 mCi/ml) was obtained from American Radiolabeled Chemicals Inc. (Saint Louis, MO). Synthetic complete medium and the yeast nitrogen base were obtained from MP Biomedica (Morgan Irvine, CA). The yeast overexpression vector pYES2/CT and *Saccharomyces cerevisiae* INVSc1 were purchased from Thermo Fisher Scientific (Carlsbad, CA). All other chemicals were obtained from either Sigma Aldrich (St. Louis, MO) or Wako (Osaka, Japan). The sequences of primers used in this study are listed in S1 Table. PLAT2 gene sequence is deposited at DDBJ as accession number LC422645.

### Culture of *A*. *limacinum*

*A*. *limacinum* was grown in GY medium (3% glucose and 1% yeast extract in 50% artificial sea water) with 0.1% vitamin mixture (vitamin B1 200 mg, vitamin B2 1 mg, and vitamin B12 1 mg/100 ml H_2_O) and 0.2% trace elements (3% EDTA di-sodium, 0.15% FeCl_3_·6H_2_O, 3.4% H_3_BO_4_, 0.43% MnCl_2_·4H_2_O, 0.13% ZnSO_4_·7H_2_O, 0.026% CoCl_2_·6H_2_O, 0.026% NiSO_4_·6H_2_O, 0.001% CuSO_4_·5H_2_O, and 0.0025% Na_2_MoO_4_·2H_2_O) at 25°C for the period indicated. Cells were harvested by centrifugation at 3,000 rpm for 5 min. Potato dextrose agar (PDA) plates (50% potato dextrose and 2% agar in 50% artificial sea water) containing 2 mg/ml of hygromycin B and 0.5 mg/ml of G418 were used to select for the *plat2*-disruption mutant (*plat2*-KO) and *plat2*-overexpression mutant (*plat2*-OE), respectively.

### Cell growth and glucose consumption of *A*. *limacinum*

After being precultured in 2 ml of GY medium for 2 days, *A*. *limacinum* was cultured in 100 ml of GY medium at 25°C with shaking. The optical density (OD) at 600 nm of the starting culture (at time 0) was 0.02. A portion of the culture of wildtype (WT) and transformants of *A*. *limacinum* was withdrawn every 48 h, and the ODat 600nm and glucose concentration were measured. The glucose concentration was quantified using Glucose CII-Test (Wako).

### Cloning of the PLAT2 gene (*plat2*) from *A*. *limacinum* F26-b

PLATs were searched for in the genome database of *Aurantiochytrium limacinum* ATCC MYA-1381 (http://genome.jgi.doe.gov/pages/blast.jsf?db=Aurli1) using human and yeast lysophospholipid acyltransferase (LPLAT) sequences as a query. The putative ORF of PLAT2 was obtained from the genomic DNA of *A*. *limacinum* F26-b by PCR using the primers 1 and 2 shown in S1 Table. The ORF of PLAT2 has no introns. The amplified PCR product was cloned into the TA cloning vector pGEM-T Easy vector system (Promega). The insert was then sequenced using the BigDye Terminator v3.1 Cycle Sequencing Kit (Applied Biosystems) and 3130 Genetic Analyzer (Applied Biosystems). The sequence of PLAT2 of *A*. *limacinum* ATCC MYA-1381 is registered as protein ID136549 in a JGI database.

### Construction and analysis of phylogenic tree of glycerolipid acyltransferases including PLATs

Sequences were aligned using ClustalW and constructed phylogenetic tree using the Maximun likelihood method using MEGA X [27, 28]. The robustness of the tree was evaluated with bootstrap test (1000 replicates) [29]. All protein sequences used for construction of phylogenetic tree are listed in S1 Appendix.

### Alignment of PLAT2 with human and drosophila GPATs

PLAT2, and human GPAT3 and 4 were aligned by the CLUSTAL algorithm using GENETYX ver. 12 [30]. The transmembrane region was estimated by TMHMM [31].

### Expression of PLAT2 in *S*. *cerevisiae*

The *plat2* expression vector was constructed by PCR using primers 3 and 4, which contained *Eco*RI and *Not*I sites. The FLAG epitope (DYKDDDDK) was also fused to the N-terminus of PLAT2 by PCR using primers 3 and 4. The fragments were then digested by *Eco*RI and *Not*I, and ligated with the yeast expression vector pYES2/CT, which is designed to work under the control of the inducible promoter GAL1.

The yeast strain INVSc1 was transformed with the expression vector using the lithium acetate method, and transformants were selected on minimal medium lacking uracil. Transformants were first grown on minimal medium containing 2% D-glucose, and then cultured on minimal medium containing 2% D-galactose at 30°C to induce *plat2* expression. After the expression of *plat2* was induced with D-galactose, cells were harvested, re-suspended in 1 ml of ice-cold 20 mM Tris-HCl, pH 7.5, containing 240 mM sucrose and 0.2 M PMSF, and crushed with 0.5 mm glass beads by a BEAD BEATER (Biospec Inc.) 5 times for 30 s. After centrifugation at 3,000 rpm for 10 min, the supernatant was used as PLAT2 enzyme.

### Preparation of cell lysate and dry cells of *A*. *limacinum*

Thraustochytrid cells were suspended in Breaking Buffer (50 mM Tris-HCl pH 7.5, 4.2 mM EDTA, 0.6 M sorbitol, and 2 mM PMSF in DMSO) with glass beads and crushed with BEADS CRUSHER μT-12 (TAITEC). After centrifugation, the supernatant was recovered and used as the cell lysate. Dry cells were prepared by freeze-drying after washing cells with distilled water.

### Determination of protein concentration

Protein concentration was measured using Pierce 660 nm Protein Assay Reagent (Thermo Scientific) and Albumin Standard (Thermo Scientific) as the standard according to the manufacturer’s instructions.

### Assay for acyltransferase activity

To measure LPLAT and glycerol-3-phosphate acyltransferase (GPAT) activity, [1-^14^C]palmitoyl-CoA and different acyl acceptors were incubated with the enzyme, and the [^14^C]-labeled products were quantified according to the methods [32, 33] after appropriate modification.

For the LPLAT assay, the reaction mixture contained 25 μM of each LPL, 1 mM EDTA, 0.01% sodium cholate, 5 μM [1-^14^C]palmitoyl-CoA (50,000 dpm/nmol), and 10 μg of protein in 100 μl of 100 mM Tris-HCl, pH 7.5. The reaction mixture was incubated at 30°C for 20 min and the reaction was then stopped by adding CHCl_3_/CH_3_OH (2:1, v/v). Total lipids were extracted and applied to a thin layer chromatography (TLC) plate, which was developed with CHCl_3_/CH_3_OH/H_2_O (65/25/4, v/v/v).

For the GPAT assay, the reaction mixture contained 2 mM MgCl_2_, 0.2 M NaCl, 8 mM NaF, 2 μM [1-^14^C]palmitoyl-CoA (50,000 dpm/nmol), 200 μM of G3P, and 20 μg of enzyme in 100 μl of 60 mM Tris-HCl buffer, pH 7.5. The assay was performed at 25°C for 45 min and the reaction was stopped by adding CHCl_3_/CH_3_OH (2:1, v/v). Lipids were extracted, and applied to a TLC plate, which was developed with CHCl_3_/CH_3_OH/CH_3_COOH/H_2_O (40/20/5/0.5, v/v/v/v).

The radioactivity of the corresponding bands was quantified using a FLA 5100 Bio-imaging analyzer (GE Healthcare).

### Generation of the *plat2-*deficient strain (*plat2*-KO)

The *plat2* KO vector, which contained the promoter, hygromycin B phosphotransferase gene (hygromycin resistance gene, HygR), and terminator, is referred to hereafter as HygR cassette. The promoter region of the *Thraustochytrium aureum* ubiquitin gene was used to drive the expression of a selection marker and HygR cassette [23, 24]. The terminator region of the SV40 virus coat protein gene was used to terminate gene transcription. The HygR cassette was added with *Bgl*II and *Sal*I sites, and inserted to the pGEM-T Easy vector (pT-HygR). Primers used to generate *plat2*-KO are shown in S1 Table.

Primers 5 and 6 were designed to insert the HygR cassette into the *plat2* ORF by homologous recombination, by which *Bgl*II and *Sal*I sites were added to the middle of the coding region. The PCR template was pT-plat2, which was the pGEM-T Easy vector containing the cloned *plat2* gene. Amplified PCR products were digested and linearized with *Bgl*II and *Sal*I. The linear fragment was treated with *E*. *coli* alkaline phosphatase (BAP) (TOYOBO Inc.) to avoid self-ligation before ligation to the HygR cassette, which was liberated from pT-HygR by digestion with *Bgl*II and *Sal*I. The newly constructed plasmid (pKO-plat2 hygR) was used as a template for PCR to prepare the linear fragment. The amplified product was cleaned with phenol-CHCl_3_-isoamyl alcohol (PCI) before transformation of *A*. *limacinum* F26-b.

*A*. *limacinum* F26-b was grown in 3 ml of GY medium at 25°C for 3 days. Cells were collected by centrifugation at 3,000 rpm for 5 min, and washed with distilled water. Cell pellets were resuspended in Nucleofector solution (Lonza) to a final concentration of 5 x 10^6^ cells /100 μl. The KO targeting vector (5 μg) was added to 100 μl of the cell suspension. The mixture was transferred into a 1-mm-gap cuvette. The cuvette was set on a GENE PULSER II (Bio-Rad), and pulsed twice (0.75 kV, 50 μF, 50Ω. The cells were added to 1 ml of fresh GY medium and incubated at 25°C overnight. Finally, all cells were transferred to hygromycin-containing PDA agar plates to select the transformants.

### Southern blot analysis of *plat2*-KO

One hundred microgram of genomic DNA was digested overnight at 37°C with *Xho*I or with *Bgl*II and *Sal*I. Digested DNA was separated on 0.7% agarose gels by electrophoresis (50 V, 60 min) and transferred to Hybond-N+ nylon membranes (GE Healthcare). Probes A and B were synthesized using a DIG DNA Labeling Kit (Roche Applied Science) according to the manufacturer’s instructions, with primers 7 and 8 (probe A), primers 9 and 10 (probe B) in S1 Table. Each membrane was incubated at a different temperature depending on the combination of probe and enzymes for DNA digestion; at 45°C for probe A and *Xho*I, and 42°C for probe B with *Bgl*II and *Sal*I. Then, all signals were detected using a DIG Nucleic Acid Detection Kit (Roche Applied Science).

### Generation of the *plat2*-overexpressing strain (*plat2*-OE)

The *plat2*-OE construct was composed of the *flag-plat2* cassette and G418 resistance cassette. First, the ORF of *plat2* was amplified by PCR using *A*. *limacinum* F26-b genomic DNA as a template and primers 11 and 12 described in S1 Table. At this time, primers were designed to add the FLAG sequence immediately after the start codon of *plat2*. *Flag-plat2* was then connected with the ubiquitin promoter and EF1α terminator by Fusion PCR using primers 13, 14, 15, and 16 to generate the *flag-plat2* cassette. After gel-purification, the PCR product was inserted into the pGEM-T (Easy) vector (Promega) using A-attachment mix (TOYOBO), and used for transformation of *Escherichia coli* JM109. The ampicillin-resistant *E*. *coli* transformants were selected, and the inserts were sequenced and confirmed. Next, the neomycin phosphotransferase (neomycin resistance gene, NeoR) cassette consisting of the EF1α promoter, NeoR, and SV40 terminator was amplified by PCR using primers 17 and 18. At this time, *Nde*I and *Spe*I sites were added to the 5 'end and 3' end of the NeoR cassette, respectively. After gel purification, the PCR product was digested with *Nde*I and *Spe*I, and fused to the *flag-plat2* cassette to prepare the *plat2-*OE construct. After transformation of *E*. *coli* with *plat2-*OE construct, ampicillin-resistant transformants were selected, and the insert was sequenced and confirmed. The *plat2*-OE cassette was then introduced into *A*. *limacinum* by electroporation using GENE PULSER Xcell (Bio-Rad) under the following conditions: resistance 200 Ω, voltage 0.75 kV, and capacitance 50 μF. After incubation in GY medium overnight, transformants were transferred to a PDA agar plate containing 0.5 mg/ml of G418. G418-resistant transformants were subjected to PCR using primers 19 and 20 and Western blotting using anti-FLAG (anti-DYKDDDDK) antibody for confirmation of *flag-plat2* expression.

### Western blotting

Western blot analysis was performed using anti-DYKDDDDK-tag antibody and anti-α-tubulin antibody. The cell lysate was mixed with 2× Sample buffer (2% SDS, 0.1 M Tris-HCl pH 7.5, 25% glycerol, appropriate amount of BPB), boiled at 95°C for 3 min, allowed to stand on ice for 5 min, and then subjected to SDS-PAGE. After electrophoresis, proteins in the gel were transferred to the Hybond P membrane (GE Healthcare) at 15 V for 30 min. The FLAG-PLAT2 was detected with anti-DYKDDDDK tag polyclonal antibody (Cell Signaling Technology) as a primary antibody, and HRP-labeled anti-rabbit-IgG (Cell Signaling Technology) as a secondary antibody. After incubation, chemiluminescence was detected using Luminata Forte Western HRP Substrate (Millipore) and Ez-Capture II (ATTO). To strip the antibody, the membrane was incubated for 30 min at 60°C using stripping buffer (50 mM Tris-HCl, pH 6.8, 2%SDS, 100 mM β-mercaptoethanol). The α-tubulin was detected with anti-α-tubulin antibody (MBL) as a primary antibody and HRP-labeled anti-mouse-IgG (Cell Signaling Technology) as a secondary antibody.

### Analysis of DG, PC, and TG using LC-ESI MS

The thraustochytrid cells were suspended in water and crushed with glass beads using BEADS CRUSHER μT-12 (TAITEC). After centrifugation, the supernatant was recovered as the cell lysate, and used for extracting lipids and measuring proteins. Total lipids were extracted from 50 μl of cell lysate by mixing and shaking at 37°C with 300 μl of CH_3_Cl/CH_3_OH (2/1, v/v) containing 10 μM lyso-PC (LPC) 13:0, 10 μM PC 22:0 (11:0/11:0), 20 μM lyso-phosphatidylethanolamine (LPE) 13:0, 20 μM phosphatidylethanolamine (PE) 24:0 (12:0/12:0), 20 μM diacylglycerol (DG) 24:0 (12:0/12:0), and 20 μM TG 36:0 (12:0/12:0/12:0) as internal standards. After centrifugation at 15,000 rpm for 5 min, 30 μl of the organic layer (lower layer) was mixed with 470 μl of 2-propanol. DG, PC, and TG were analyzed by LC-ESI MS according to the method described by Ikeda et al [34]. LC-ESI MS was performed using a HPLC system (Agilent Technologies) coupled to a MS apparatus (3200 QTRAP; AB Sciex) [20]. A binary solvent gradient with a flow rate of 200 μl/min was used to separate DG, PC, and TG by reverse-phase chromatography using InerSustain C18 (2.1 x 150 mm, 5 μm; GL Sciences). The gradient was started with 3% B (2-propanol with 0.1% formic acid and 0.028% ammonia) in buffer A (acetonitrile/CH_3_OH/distilled water, 19/19/2, v/v/v containing 0.1% formic acid and 0.028% ammonia) and was maintained for 3 min. The gradient reached 40% B for 21 min, then 70% B for 1 min, and was maintained for 7 min. The gradient was returned to the starting conditions for 1 min and the column was equilibrated for 7 min before the next run. For phospholipid analysis, precursor ion scan at *m/z* 184, corresponding to the phosphocholine head group, and neutral loss scan at *m/z* 141, corresponding to the phosphoethanolamine head group, were used to identify the molecular species of PC and PE, respectively. For neutral lipid analysis, neutral loss scans at *m/z* 273, 345, and 347 were used to identify DG and TG containing palmitic acid and DHA, respectively. The structures of phospholipids and neutral lipids were confirmed by MS/MS analysis. Lastly, DG, PC, and TG were measured using multiple reaction monitoring (MRM). Stable isotope-labeled palmitic acid (d31-16:0) (Cambridge Isotope Laboratories) was added to the medium of *A*. *limacinum* at a final concentration of 11.5 μM to trance the synthesis of PC and TG *in vivo*. Cells were collected after adding d31-16:0 for 10, 20, 40, 60, 120, 240, or 480 min. Lipids were extracted from cell lysates as described above. The d31-16:0-containing PC and TG were measured using MRM.

### Analysis of LPA using LC-ESI MS

Total lipids were extracted from 50 μl of cell lysate by mixing and shaking at 37°C with 300 μl ofCHCl_3_/CH_3_OH (2/1, v/v) containing 20 μM LPE 13:0 as an internal standard. To the 150-μl organic layer obtained after centrifugation, 20 μl of trimethylsilyl diazomethane solution (2.0 M in hexanes, Sigma Aldrich) was added for methylation of the sample [35]. After vortexing for 30 s, methylation was performed at 50°C for 10 min. The reaction was terminated by adding 6 μl of glacial acetic acid, and then subjected to LC-ESI MS. LC-ESI MS was carried out using an HPLC system (Agilent Technologies) coupled to a MS apparatus (3200 QTRAP). A binary solvent gradient with a flow rate of 200 μl/min was used to separate LPA and PA by reverse-phase chromatography using InerSustain C18 (2.1 x 150 mm, 5 μm; GL Sciences). The gradient was started with 3% B (2-propanol with 0.1% formic acid and 0.028% ammonium) in buffer A (acetonitrile/CH_3_OH/H_2_O, 19/19/2, v/v/v, containing 0.1% formic acid and 0.028% ammonium) and was maintained for 3 min. The gradient reached 40% B for 21 min, then 70% B for 1 min, and was maintained for 7 min. The gradient was returned to the starting conditions for 1 min and the column was equilibrated for 7 min before the next run. Neutral loss scan at 126, corresponding to methylated LPA, was used to identify the molecular species of LPA. The structure of LPA was confirmed by MS/MS analysis.

### Statistical analysis

Data were collected from at least three separate experiments and are reported as the mean ± standard deviation (SD). Statistical analysis was performed by a 2-tailed Student’s t test using a GraphPad Prism 6 for 2 group comparison. *, *p* < 0.05; **, *p*< 0.01.

## Results and discussions

### Molecular cloning and characterization of PLAT2 from *Aurantiochytrium limacinum* F26-b

**Fig 1:**
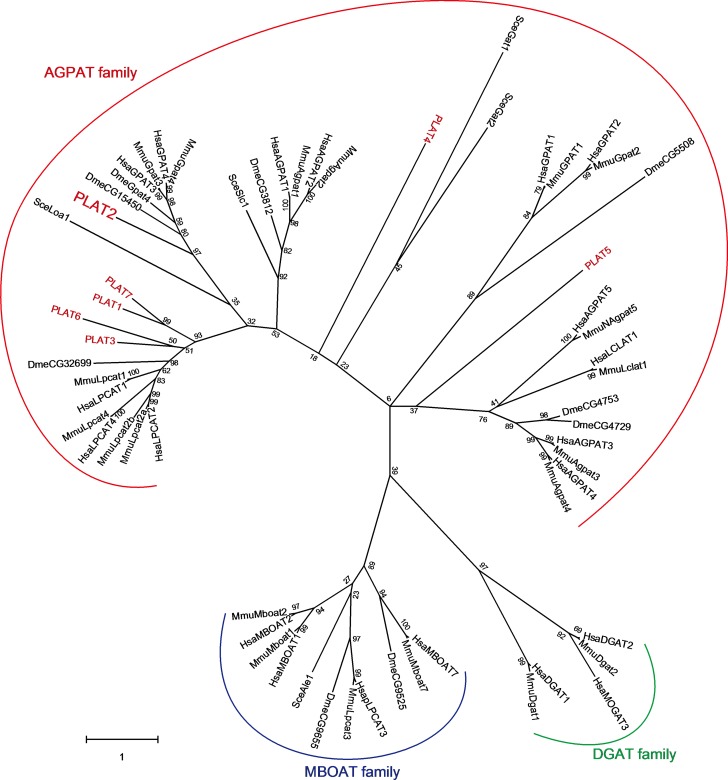
Phylogenetic tree of glycerolipid acyltransferases including PLATs. Using CLUSTALW, the deduced amino acid sequences of LPLATs and DGATs from human, mouse, drosophila, and budding yeast, and PLATs from *A*. *limacinum* were subjected to multiple alignment analysis. Sequences of these enzymes were obtained from NCBI and JGI (https://genome.jgi.doe.gov/Aurlil/Aurli.home.html, US Department of Energy) databases, respectively. We analyzed the relationship of each protein with Maximum Likelihood method and JTT matrix-based model using MEGA X (27, 28). The percentage shown next to branches was the robustness of the tree which was evaluated with the bootstrap test (1000 replicates) (29). Only numbers of 50% or more are displayed. All protein sequences used for construction of phylogenetic tree are listed in S1 Appendix. Hsa, *Homo sapiens*; Mmu, *Mus musculus*; Sce, *Saccharomyces cerevisiae*; Dme, *Drosophila melanogaster*; Aku, *Aplanochytrium kerguelense*; Sag, *Schizochytrium aggregatum*; Hfe, *Hondaea fermentalgiana*; AGPAT, 1-acylglycerol-3-phosphate *O*-acyltransferase; MBOAT, membrane bound *O*-acyltransferase; DGAT, diacylglycerol acyltransferase.

Lysophospholipid acyltransferase (LPLAT) homologues were surveyed in the genome database of *A*. *limacinum* ATCC MYA-1381 (type strain) using budding yeast and human LPLATs as queries. As a result, we found several putative gene products as LPLAT homologues in *A*. *limacinum*, which we tentatively designated as PLAT1~7 in this study. Based on the phylogenetic tree, all PLATs found in thraustochytrids were in the AGPAT family, and PLAT2 was selected as a candidate for GPAT because it was evolutionary close to mammalian and *Drosophila* GPATs. It is worth noting that PLAT2 homologues are also found in other thraustochytrids (Fig 1).

**Fig 2:**
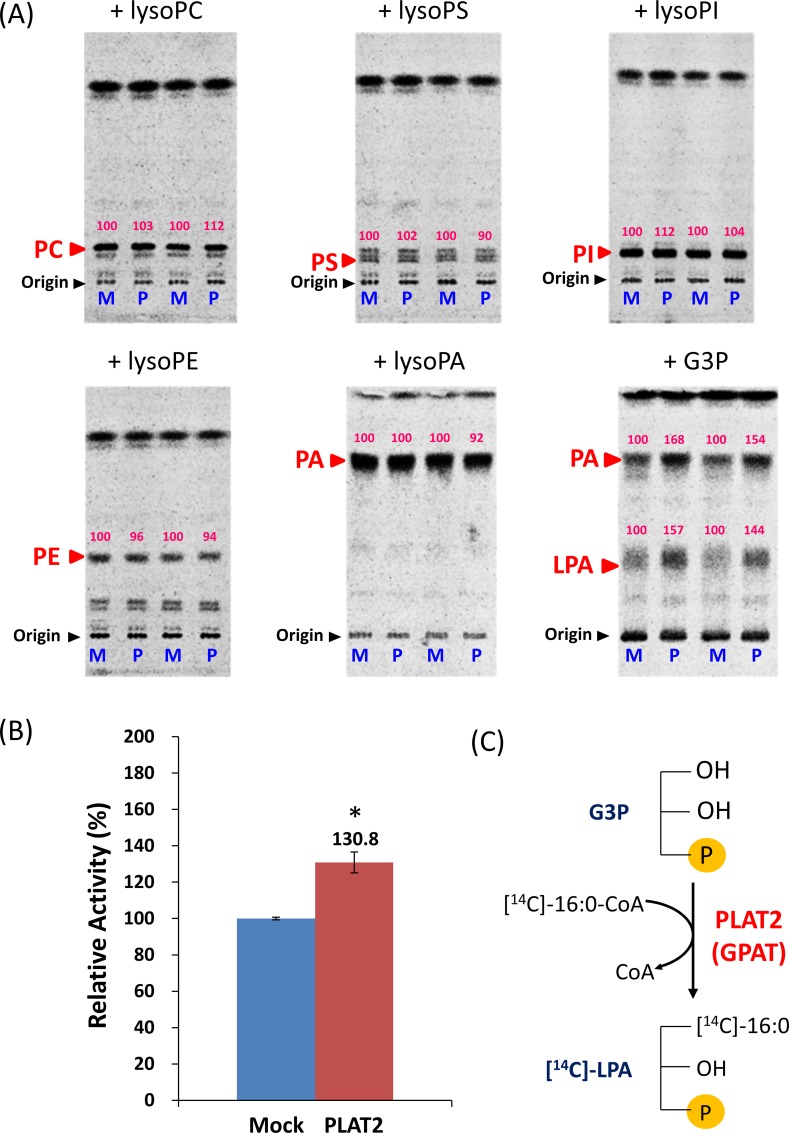
PLAT2 activity expressed in budding yeast. (A), LPLAT activity was measured using LPA, LPC, LPS, LPI, or LPE as an acceptor substrate, ^14^C-labeled palmitoyl-CoA as a donor substrate, and cell lysate as an enzyme, as described in Materials and Methods. The reaction was conducted at 30°C for 20 min. Total lipids were extracted after terminating the reaction by adding 500 μl of CHCl_3_/CH_3_OH (2/1, v/v), and was then applied to a thin layer chromatography (TLC) plate, which was developed with CHCl_3_/CH_3_OH/H_2_O (65/25/4, v/v/v). GPAT activity was measured using G3P as an acceptor substrate, ^14^C-labeled palmitoyl-CoA as a donor substrate, and cell lysate as an enzyme, as described in Materials and Methods. The assay was performed at 25°C for 45 min and terminated by adding 500 μl of CHCl_3_/CH_3_OH (2/1, v/v). The reaction mixture was loaded on the TLC plate and developed with CHCl_3_/CH_3_OH/acetic acid/H_2_O (40/20/5/0.5, v/v/v/v). The radioactivity of the corresponding bands was quantified using a FLA 5100 Bio-imaging analyzer (GE Healthcare). Numbers on TLC plates indicate the relative activity when the enzyme activity of mock used as a control is taken as 100. M, lysate of mock transfectant of *S*. *cerevisiae*; P, lysate of PLAT2-expressing *S*. *cerevisiae*. (B), GPAT activity was measured independently of (A), but by the same method. Data represent the mean±SD (n = 3). *, *p* < 0.05; **, *p*< 0.01 (*versus* WT). (C), Schematic illustration showing the transfer of [^14^C]palmitic acid from [^14^C]palmitoyl CoA to G3P, generating [^14^C]LPA by PLAT2.

PLAT2 was cloned from *A*. *limacinum* F26-b, which is genetically close to *A*. *limacinum* ATCC MYA-1381 [19], and subjected to heterologous expression in *Saccharomyces cerevisiae*. Then, LPLAT activity was measured using different lysophospholipids and [1-^14^C]-palmitoyl-CoA as substrates. As shown in Fig 2A, the lysate of PLAT2-expressing yeast exhibited almost no LPLAT activity toward LPC, lyso-PS (LPS), lyso-phosphatidylinositol (LPI), LPE, or LPA; however, the generation of [^14^C]-LPA increased when G3P was added to the lysate of PLAT2-expressing yeast compared with the mock transfectant. [^14^C]-PA also increased when G3P was added to the reaction (Fig 2A, right panel in the 2nd row); however, this was likely a secondary effect due to the increase in [^14^C]-LPA by PLAT2 because generation of [^14^C]-PA did not increase when LPA was added to the reaction (Fig 2A, center panel in the 2nd row). GPAT activity of lysate of PLAT2-expressing *S*. *cerevisiae* was significantly higher than that of mock transfectant (Fig 2B). These results indicate that PLAT2 is a GPAT that converts G3P to LPA (Fig 2C).

**Fig 3:**
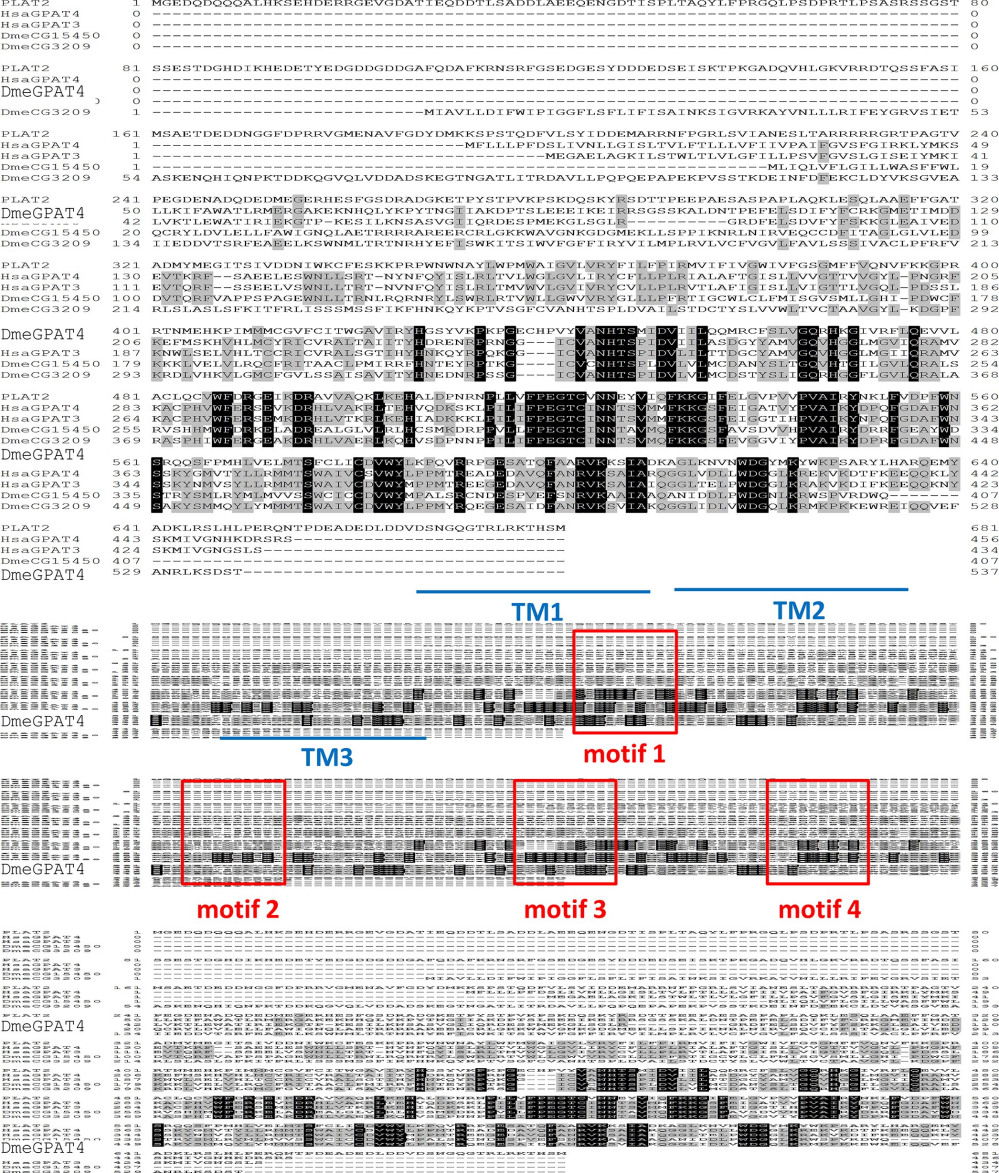
Alignment of PLAT2 with human and drosophila GPATs. Identical amino acids in two or three sequences are indicated by black letters on gray boxes and white letters on black boxes, respectively. TM1, 2, and 3 indicate the transmembrane regions, and motifs 1, 2, 3, and 4 indicate the motifs conserved in several GPATs (Table 1).

The open reading frame (ORF) of the PLAT2 gene (*plat2*) encoded a putative 77.2-kDa protein containing three possible transmembrane domains and four acyltransferase motifs (Fig 3). These characteristic motifs were conserved in GPATs of different origins [36] (Table 1).

**Table 1:** Acyltransferase motifs conserved in GPATs.

Protein	Motif 1	Motif 2	Motif 3	Motif 4
SceGat2p	GAPHANQFIDPA	GGIPVPRIQ	FPEGGSHDR	VAVVPCGLHY
SceGat1p	AAPHANQFVDPV	MAIGVVRPQ	FPEGGSHDR	VKIVPCGMNY
DmeGPAT1	VPLHRSHLDYIMV	LGAFFIKRKI	FFIEGGRTR	ALLVPVSVNYE
HsaGPAT1	LPVHRSHIDYLLL	LGGFFIRRRL	IFLEGTRSR	ILIIPVGISYD
MmuGPAT1	LPVHRSHIDYLLL	LGGFFIRRRL	IFLEGTRSR	ILVIPVGISYD
HsaGPAT2	LSTHKTLLDGILL	LGGLFLPPEA	IFLEEPPGA	ALLVPVAVTYD
MmuGPAT2	LSTHKSLLDGFLL	LGGLFLPPEV	IFLEEPPGS	ATLVPVAIAYD
MmuGPAT3	VANHTSPIDVLIL	PHVWFERSEI	IFPEGTCIN	GTIYPVAIKYN
HsaGPAT3	VANHTSPIDVLIL	PHVWFERSEM	IFPEGTCIN	GTIHPVAIKYN
DmeGPAT4	VANHTSPIDVLVL	PHIWFERGEA	IFPEGTCIN	GVIYPVAIKYD
MmuGPAT4	VANHTSPIDVIIL	PHVWFERSEV	IFPEGTCIN	ATVYPVAIKYD
HsaGPAT4	VANHTSPIDVIIL	PHVWFERSEV	IFPEGTCIN	ATVYPVAIKYD
PLAT2	VANHTSMIDV	QCVWFDRGEI	VFPEGTCVN	VPVVPVAIRYN

The underlined letters indicate amino acid residues that are conserved in each motif. Sce; *Saccharomyces cerevisiae*, Dme; *Drosophila melanogaster*, Mmu; *Mus musculus*, Hsa; *Homo sapiens*.

Alignment of PLAT2 with human GPAT3 (HsaGPAT3) and GPAT4 (HsaGPAT4), and *Drosophila* GPAT4 (DmeGpat4, CG3209) revealed that PLAT2 is highly homologous to these GPATs (Fig 3, Fig 1); however, the N-terminal region of PLAT2 was longer than those of human and *Drosophila* GPAT4 [37, 38].

### Generation and validation of *plat2*-disruption mutant (*plat2*-KO) and *plat2*-overexpression mutant (*plat2*-OE)

**Fig 4:**
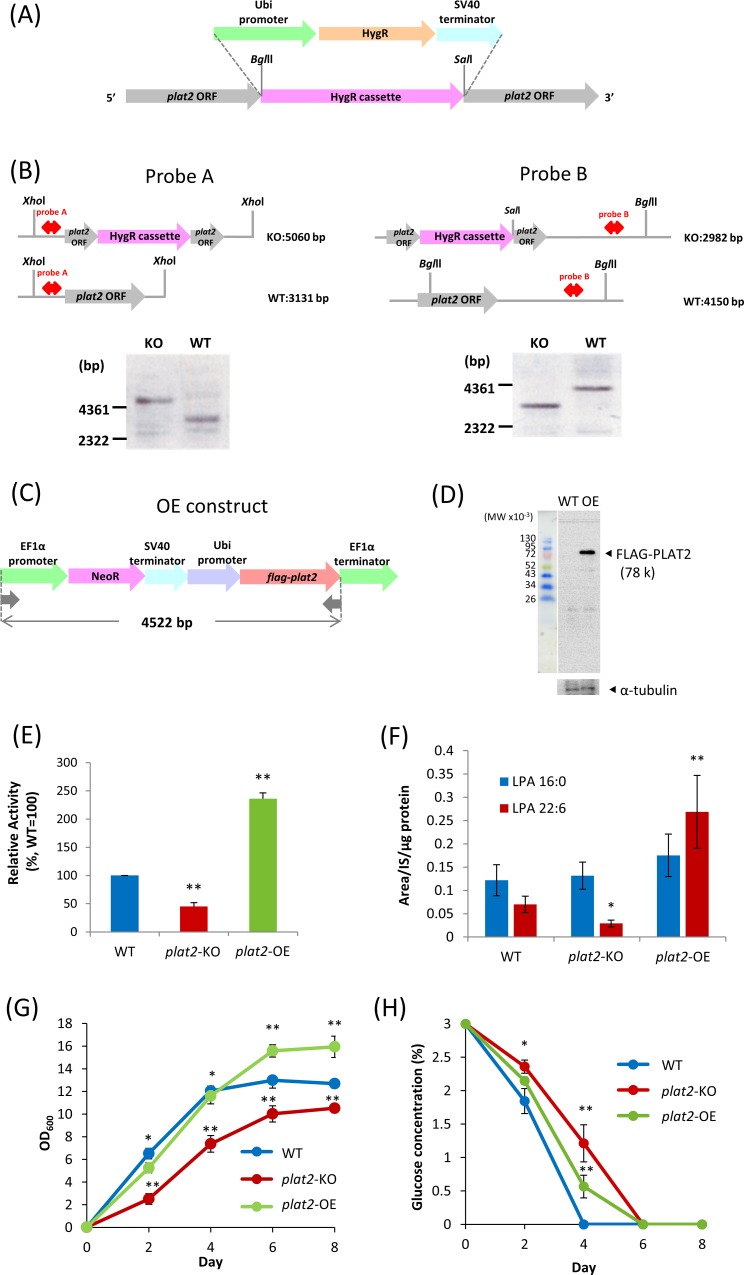
Overexpression and deletion of *plat2* gene in *A*. *limacinum*. (A), Strategy for disruption of *plat2* by hygromycin B phosphotransferase gene (hygromycin resistance gene, HygR) cassette. (B), Southern blot showing the deletion of *plat2* in *plat2*-KO (KO). WT, *A*. *limacinum* F26-b wildtype. (C), Strategy for overexpression of PLAT2 with the FLAG-tag at the C-terminal. NeoR represents neomycin phosphotransferase (neomycin resistance gene). (D), Western blot showing the expression of FLAG-tagged PLAT2 (FLAG-PLAT2) in *plat2*-OE. (E), GPAT activity of WT, *plat2*-KO, and *plat2*-OE. GPAT activity was measured by the method described in Materials and Methods using cell lysates from each strain at day 2. (F), LPA amounts were measured by LC-ESI MS, as described in Materials and Methods, using cell lysates from each strain at day 2. Data in E and F represent the mean±SD (n = 3). *, *p* < 0.05; **, *p*< 0.01 (*versus* WT). (G), Cell growth of WT, *plat2*-KO, and *plat2*-OE. Each strain was cultured in 100 ml of GY medium with shaking. Blue, red, and green circles represent OD at 600 nm of WT, *plat2*-KO, and *plat2*-OE, respectively. (H), Glucose consumption of WT, *plat2*-KO, and *plat2*-OE. Blue, red, and green circles represent the concentration (%) of glucose in the medium of WT, *plat2*-KO, and *plat2*-OE, respectively. Data in Fig G and H represent the mean±SD (n = 3). * represents p<0.05; **, *p*< 0.01 (*versus* WT).

We generated *plat2*-KO and *plat2*-OE of *A*. *limacinum* F26-b in order to investigate the functions of PLAT2 *in vivo*. The strategies for the disruption and overexpression of *plat2* are shown in Fig 4A and 4C, respectively. Southern and Western blotting confirmed that *plat2* was successfully disrupted in *plat2*-KO (Fig 4B), and that FLAG-tagged PLAT2 was correctly expressed in *plat*2-OE (Fig 4D). GPAT activity was found to have decreased in *plat2*-KO and increased in *plat2*-OE when the cell lysates of corresponding mutants were used for enzyme assay (Fig 4E). The amounts of LPA of WT, *plat2*-KO, and *plat2*-OE were examined by LC-ESI MS. As a result, we found that the amount of DHA-containing LPA (LPA 22:6) increased in *plat2*-OE and decreased in *plat2*-KO, as compared with WT (Fig 4F). On the other hand, the amount of LPA16:0 did not change under the conditions used when *plat2* was disrupted or overexpressed (Fig 4F). These results indicate that PLAT2 functions as a GPAT that incorporates DHA into G3P to generate LPA 22:6 *in vivo*. However, it remains unclear whether PLAT2 prefers DHA-CoA over 16:0-CoA or whether PLAT2 can more easily access the DHA-CoA pool than that of 16:0-CoA *in vivo*. The *in vitro* experiment using purified PLAT2 may provide an answer for the former query, but we have yet successfully purified PLAT2 from the cell lysates of *A*. *limacinum* and *S*. *cerevisiae* after overexpression of *plat*2 because of the difficulty in solubilizing the membrane spanning protein with GPAT activity.

The cell growth of *plat2-*KO *and plat2-*OE transfectants was compared with that of WT by measuring the turbidity (OD at 600 nm) of the culture fluid. The growth curve of *plat2*-OE was almost the same as that of WT until day 4, but WT reached a plateau slightly faster than *plat2*-OE. The cell growth of *plat2*-KO was significantly suppressed during the course of culture (Fig 4G). Consistent with the growth curves, glucose consumption by WT was faster than the *plat2*-OE or *plat2*-KO (Fig 4H).

### Effects of deletion and overexpression of PLAT2 on glycerolipid synthesis

**Fig 5:**
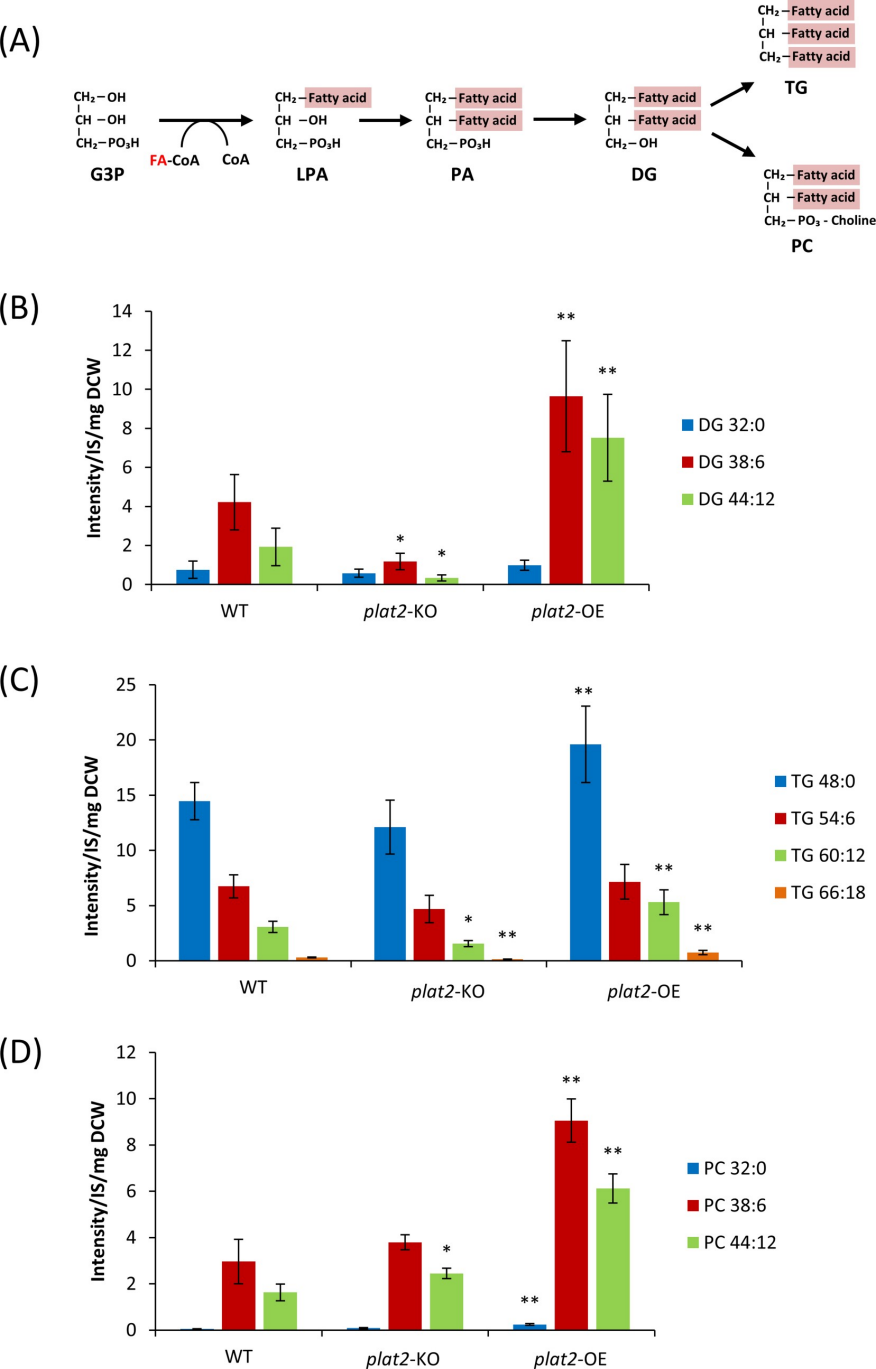
Effects of overexpression and deletion of PLAT2 on glycerolipid synthesis. (A), Schematic diagram showing the assumed glycerolipid synthesis pathway in *A*. *limacinum*. (B, C, D), Amounts of major molecular species of DG (B), TG (C), and PC (D) in WT, *plat2*-KO, and *plat2*-OE cultured at day 4 in 100 ml of GY medium with shaking. Individual lipid species were quantified by LC-ESI MS as shown in Materials and Methods, and represented as the peak intensity/each internal standard/ mg of the dry cell weight. All data represent the mean±SD (n = 3). * represents p<0.05; **, *p*< 0.01 (*versus* WT).

The presumed *de novo* synthesis pathway of glycerolipids in thraustochytrids is shown in Fig 5A. This pathway starts from LPA synthesis by GPAT. As shown in Fig 4F, overexpression and depletion of *plat2* resulted in an increase and decrease of LPA22:6. Then, we examined the effects of manipulation of *plat2* on the production of several molecular species of TG, PC, and their precursor DG. We measured the amounts of these lipids in WT, *plat2*-KO, and *plat2*-OE using LC-ESI MS. As shown in Fig 5B, overexpression of PLAT2 significantly increased the amount of DHA-containing DG (DG 38:6 and DG 44:12) but not DHA-free DG (DG 32:0). This result is well consistent with the result of Fig 4F, which indicated that overexpression of PLAT2 increased the amount of LPA 22:6 but not LPA 16:0. We found that DHA-rich TG (TG 60:12, TG 66:18) and DHA-containing PC (PC 38:6, PC 44:12) also significantly increased in *plat2*-OE (Fig 5C and 5D). In contrast, DHA-rich TG, such as TG 60:12 and TG 66:18, and their precursor, DG 38:6 and DG 44:12, decreased by deletion of *plat2* gene, while PC 38:6 and PC 44:12 did not decrease in *plat2*-KO. This is probably because PC synthesis is a priority even in *plat2*-KO. The effect of disruption of *plat*2 on the production of glycerolipids was relatively smaller than that of overexpression of enzyme, possibly due to the presence of other GPAT(s) capable of compensating for the lack of PLAT2 in *A*. *limacinum*. Another possible explanation is that *A*. *limacinum* may possess the acyl dihydroxyacetone phosphate (acyl-DHAP) pathway by which G3P is converted to LPA via DHAP and acyl-DHAP [39].

Overexpression of GPAT4 in HepG2 cells increased the TG content by 20% [40]. We found in this study that overexpression of PLAT2 increased the amount of not only TG (Fig 5C) but also PC (Fig 5D) in *A*. *limacinum*. In particular, DHA-rich TG, such as TG 60:12 and TG 66:18, and DHA-rich PC, such as PC 44:12, increased in *plat 2*-OE. These results indicated that PLAT2 contributes the biosynthesis of DHA-containing glycerolipids in the glycerolipid synthesis pathway of *A*. *limacinum*, especially DHA-rich TG and PC. Collectively, this study demonstrated that the generation of LPA 22:6 by PLAT2 is the limiting step for the production of DHA-rich TG and PC in *A*. *limacinum*.

### Conversion of DG 32:0 to TG 48:0 and PC 32:0

**Fig 6:**
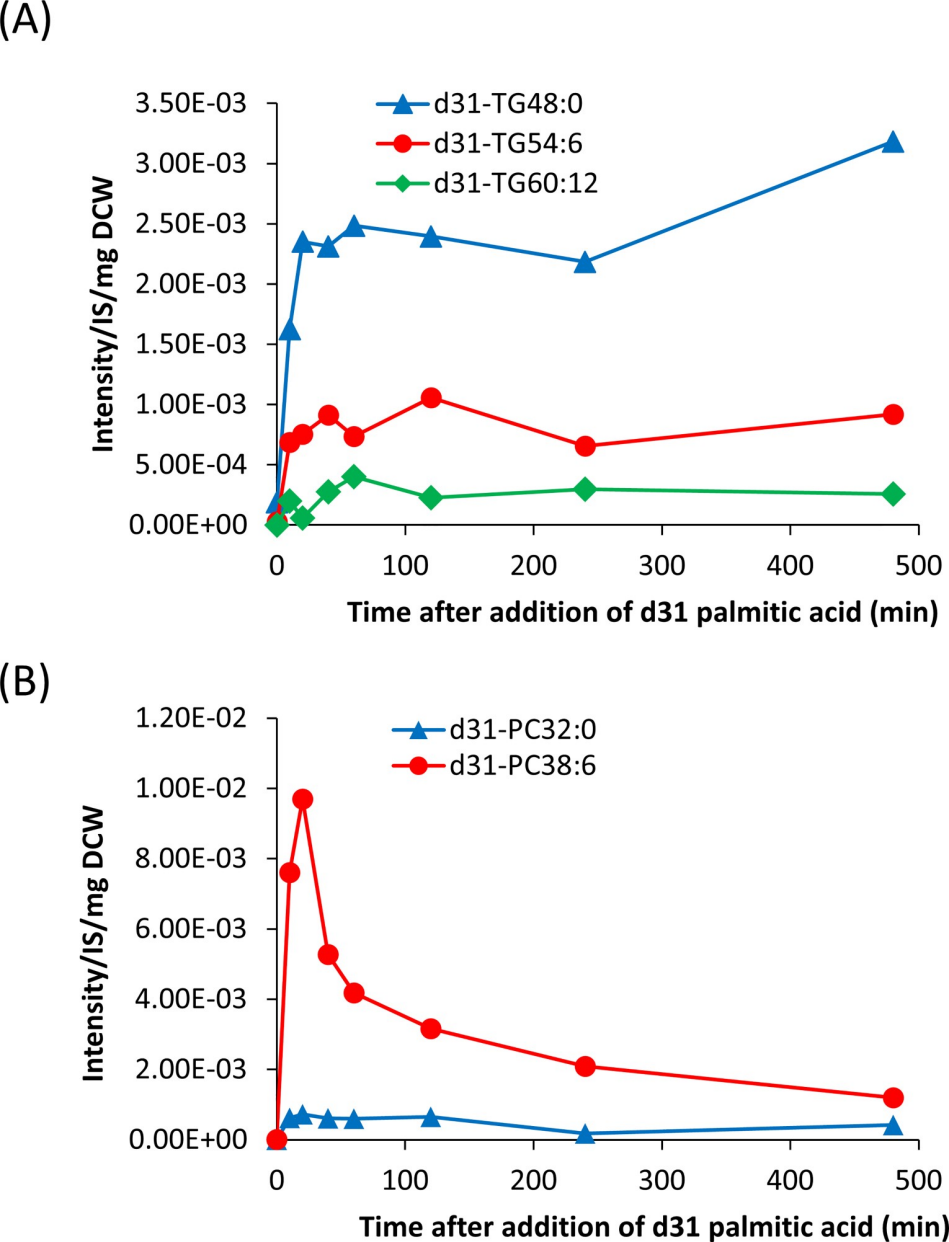
Monitoring of TG and PC syntheses using stable isotope-labeled palmitic acid. Incorporation of stable isotope-labeled 16:0 (d31-palmitic acid) into different molecular species of TG (A) and PC (B). Stable isotope-labeled palmitic acid (d31-16:0) (Cambridge Isotope Laboratories) was added to the medium of *A*. *limacinum* at a final concentration of 11.5 μM to trance the synthesis of PC and TG *in vivo*. Cells were collected after adding d31-16:0 for 10, 20, 40, 60, 120, 240, or 480 min. Lipids were extracted from cell lysates as described above. Lipids were extracted from dry cells, and d31-palmitic acid-containing PC and TG were measured by MRM with LC-ESI MS.

We found by LC ESI-MS analysis that the amount of DHA-free TG (TG 48:0) was much higher than that of DHA-free PC (PC 32:0) (Fig 5C *vs* 5D), suggesting that DG 32:0 is preferentially converted to TG 48:0 but not PC 32:0. To confirm this hypothesis, we explored the time-course conversion of stable isotope-labeled palmitic acid (d31-16:0) to TG and PC in *A*. *limacinum* (Fig 6). The d31-16:0 added to the culture of *A*. *limacinum* was incorporated rapidly into TG 48:0 (d31-TG 48:0), compared with TG 54:6 (d31-TG 54:6) and TG 60:12 (d31-TG 60:12), suggesting d31-16:0 was preferentially converted to TG 48:0 through DG 32:0. On the other hand, the incorporation of d31-16:0 into PC 32:0 (d31-PC 32:0) was much slower than that into PC 38:6 (d31-PC 38:6). These results suggested that DG 32:0 was preferentially converted to TG 48:0, compared to PC 32:0.

### Significance of PLAT2 in DHA-rich glycerolipid synthesis

**Fig 7:**
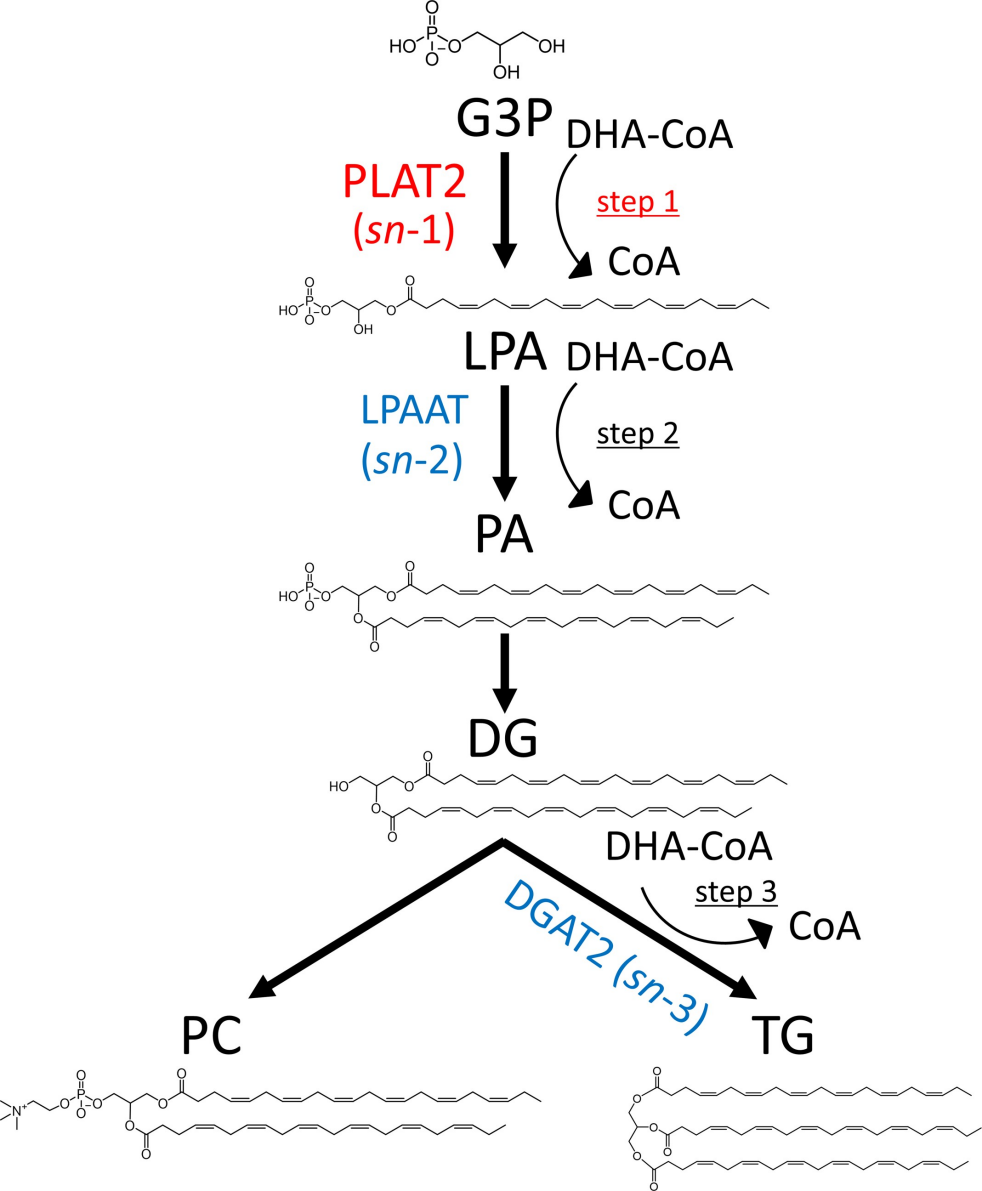
Hypothetical diagram showing the possible steps for incorporation of DHA into PC and TG in *de novo* synthesis. DHA may be incorporated in *de novo* syntheses of LPA (step 1), PA (step 2), or TG (step 3). For the generation of two DHA-containing PC (PC 44:12), DHA should be incorporated into the glycerol backbone at steps 1 and 2. On the other hand, DHA should be incorporated at two of the three steps (1, 2, and 3) for the generation of two DHA-containing TG (TG 60:12) and at all three steps for three DHA-containing TG (TG 66:18). Thus, DHA incorporation into the glycerol backbone of G3P by PLAT2 (step 1) is important for generation of DHA-rich PC and TG.

A hypothetical diagram showing the possible steps for incorporation of DHA into glycerol backbone of glycerolipids in *de novo* synthesis is presented in Fig 7. *De novo* synthesis of glycerolipids starts with the reaction of transferring fatty acid from acyl-CoA to the *sn-1* position of G3P to generate LPA [41] (step 1 in Fig 7). This reaction is mainly catalyzed by PLAT2 in *A*. *limacinum*, as demonstrated in the present study. LPA is then converted to PA by LPAAT (1-acylglycerol-3-phosphate *O*-acyltransferase), which catalyzes the transfer of a fatty acid from acyl-CoA to the *sn-*2 position of LPA (step 2) [42]. Subsequently, PA is dephosphorylated by a PA phosphatase to generate DG, which is a precursor for TG and PC synthesis. DG is converted to either TG (step 3) by diacylglycerol acyltransferase 2 (DGAT2) [43] or PC by choline phosphotransferase [44]. The fatty acyl chains of PC may be remodeled by LPCAT, *e*.*g*. PLAT1 in *A*. *limacinum* [19]. Collectively, DHA can be incorporated into the glycerol backbone at three steps in *de novo* synthesis, i.e., LPA synthesis (step 1), PA synthesis (step 2), or TG synthesis (step 3). For the generation of DHA-rich PC (PC 44:12), DHA should be theoretically incorporated during both LPA and PA syntheses (steps 1 and 2), or LPA synthesis (step 1) and PC remodeling. On the other hand, DHA should be theoretically incorporated at two of three steps (two of steps 1, 2, and 3) for TG 60:12 synthesis and at all three steps (steps 1, 2, and 3) for TG 66:18 synthesis. Thus, in any of the above cases, DHA incorporation by PLAT2 into G3P (step 1) is important for the synthesis of DHA-rich TG and PC in *A*. *limacinum*. The result that DHA-rich TG and PC increased significantly with PLAT2 overexpression strongly supports this hypothesis.

## Conclusion

PLAT2 is a rate-limiting enzyme in DHA-rich glycerolipid synthesis in *A*. *limacinum*. The mechanism by which DHA is incorporated into the glycerol backbone at the early step of glycerolipid synthesis by PLAT2 is reasonable for thraustochytrids, which produce DHA-rich glycerolipids.

## Supporting information

S1 Table Primer list used in this study. The number of primers in this table corresponds to that in the Materials and methods. (F) and (R) indicate the forward and reverse primers, respectively.
(DOCX)

S1 Appendix Accession numbers and sources of protein sequences used for constructing phylogenetic tree of glycerolipid acyltransferases including PLATs (Fig 1). (XLSX)
