# Supplementary material for: The glycerol-3-phosphate acyltransferase PLAT2 functions in the generation of DHA-rich glycerolipids in Aurantiochytrium limacinum F26-b
Source: PLoS One. 2019 Jan 30;14(1):e0211164. doi: 10.1371/journal.pone.0211164 (PMC6353168; doi:10.1371/journal.pone.0211164)
Supplement: S1 Table — The number of primers in this table corresponds to that in the Materials and methods. (F) and (R) indicate the forward and reverse primers, respectively. (DOCX) [file pone.0211164.s001.docx]

**S1 Table. Primer list used in this study**

|  | Purpose | | Sequence (5’-3’) |
| --- | --- | --- | --- |
| 1 | *plat2* cloning | Amplification of *plat2* ORF (F) | ATGGGTGAAGATCAAGATCAACAGC |
| 2 |  | Amplification of *plat2* ORF (R) | CTACATAGAGTGGGTTTTGCGAAGGCG |
| 3 | *plat2* expression (*S. cerevisiae)* | Amplification of *flat-plat2* ORF (Underline shows the *plat2* sequence, and the double line shows flag tag sequence) (F) | CGTGAATTCATGGATTACAAGGATGACGATGACAAGGGTGAAGATCAAG |
| 4 |  | Amplification of *flat-plat2* ORF (R) | ATAATAATGCGGCCGCATCATAGAGTGGG |
| 5 | *plat2* knockout | Amplification of *plat2* knock-out cassette (F) | GCAGATCCAGATCTCCCAAAAATTATCATCC |
| 6 |  | Amplification of *plat2* knock-out cassette (R) | GTCGACAAGTGTTTCGAAAGTAAGAAACC |
| 7 | *plat2* knockout southern blot | Amplification of probe A (F) | AAACCGCTTTCATTCTGACGCTTGC |
| 8 |  | Amplification of probe A (R) | ATTGCAAGAACTGCAAGCCTCTC |
| 9 |  | Amplification of probe B (F) | CACCCGATTATAAGGAGTGTCGCTTGC |
| 10 |  | Amplification of probe B (R) | GAACAGCTGCGTAATCTTGCAAACGGT |
| 11 | *plat2* expression (*A. limacinum*) | Amplification of *flag-plat2* ORF (Underline shows the *plat2* sequence, and the double dline shows *flag* tag sequence) (F) | GCAACACTAGCCAACATGGATTACAAGGATGACGATGACAAGGGTGAAGATCAAGATC |
| 12 |  | Amplification of flag-*plat2* ORF (Underline shows the plat2 sequence) (R) | CATAGCCGGCGCGGATCCTACATAGAGTGGG |
| 13 |  | Amplification of ubiquitin promoter (Underline shows the ubiquitin promoter sequence) (F) | CATAGATCTGAGAGCGTTTGCTTCGAGCCGC |
| 14 |  | Amplification of ubiquitin promoter (Underline shows the ubiquitin promoter sequence) (R) | CATCCTTGTAATCCATGTCGCTTGCTGCTGCTGGTG |
| 15 |  | Amplification of EF1a terminator (Under line shows the EF1a terminator sequence) (F) | CAAAACCCACTCTATGTAGGATCCGCGCCGGCTATG |
| 16 |  | Amplification of EF1a terminator (Under line shows the EF1a terminator sequence) (R) | CATAGATCTCGAAAGACGGGCCGTAAGGACG |
| 17 |  | Amplification of NeoR expression cassette (Under line shows the EF1a promoter sequence) (F) | CAACATATGAAGCTTCGCGCATTCGTCCTC |
| 18 |  | Amplification of NeoR expression cassette (Under line shows the EF1a promoter sequence) (R) | CATACTAGTCGAATTCGAGCTCGGTACCC |
| 19 |  | Amplification of *plat2* overexpression cassette (F) | GAGCTCTCCCATATGAAGCTTCGCGC |
| 20 |  | Amplification of *plat2* overexpression cassette (R) | GATATCATAGATCTCGAAAGACGGGCCG |

The number of primers in this table corresponds to that in the Materials and methods.

(F) and (R) indicate the forward and reverse primers, respectively.
